# Comparison of updated birth weight, length and head circumference charts by gestational age in China with the INTERGROWTH-21st NCSS charts: a population-based study

**DOI:** 10.1007/s12519-022-00631-4

**Published:** 2022-10-28

**Authors:** Ya-Qin Zhang, Hui Li, Xin-Nan Zong, Hua-Hong Wu

**Affiliations:** Department of Growth and Development, Capital Institute of Pediatrics, No. 2, Ya Bao Road, Chaoyang District, Beijing, China

**Keywords:** Birth length, Birth weight, Growth charts, Head circumference, Newborns

## Abstract

**Background:**

INTERGROWTH-21st Newborn Cross-Sectional Study (NCSS) charts were established and recommended for global application. However, whether one international reference is appropriate for all populations is still unclear. We aim to compare the updated Chinese birth size charts by gestational age with INTERGROWTH-21st NCSS charts.

**Methods:**

A cross-sectional survey was carried out, and the birth weight, length and head circumference of 24,375 infants born after uncomplicated pregnancies at gestational age ranging from 24^+0^ to 42^+6^ weeks were measured in 13 cities in China from 2015 to 2018. Growth charts were constructed. The measurements of all these infants were evaluated by the methods of calculating their *Z* scores using the INTERGROWTH-21st standards. The prevalence of small for gestational age (SGA) and large for gestational age (LGA) based on birth weight was analyzed using Chinese charts and INTERGROWTH-21st charts.

**Results:**

The mean *Z* scores were 0.10 for birth weight, 0.35 for length and − 0.02 for head circumference. Compared to the INTERGROWTH-21st charts, the Chinese birth weight percentile curves were higher except for the 90th percentile at 29–37 weeks gestational age, and the length percentile curves were higher after 33 weeks gestational age, while the 10th percentile of the head circumference was lower and the other percentiles were similar. The prevalence of SGA was 10.1% [95% confidence interval (CI) = 9.7%–10.5%] using the Chinese birth weight chart and 6.5% (95% CI = 6.2%–6.8%) using the INTERGROWTH-21st birth weight chart. The prevalence of LGA was 9.9% (95% CI = 9.5%–10.2%) and 8.2% (95% CI = 7.9%–8.6%) using the Chinese and INTERGROWTH-21st birth weight charts, respectively.

**Conclusions:**

Chinese birth size charts based on infants born after uncomplicated pregnancies were different from the INTERGROWTH-21st charts. Differences in the classification of newborns by the two charts should receive attention, and whether the application of INTERGROWTH-21st in Chinese newborns will lead to misclassification needs to be validated in future clinical practice.

## Introduction

The effective identification of high-risk newborns with abnormal growth plays an important role in health risk prediction, prognosis assessment and early intervention [1]. Birth size charts by gestational age, including birth weight, birth length and head circumference, are easy-to-use tools for the classification of newborns and their early growth monitoring and health care [2–5].

Birth size charts by gestational age are usually established based on birth registration data [6–11]. It is noted that the source data of these charts cannot exclude some high-risk newborns with abnormal intrauterine growth, which may affect the growth assessment. Therefore, establishing birth size charts by gestational age based on low-risk newborns without intrauterine growth restriction is proposed [12], and they may be helpful for more effectively identifying neonates with abnormal growth and adverse health outcomes [13]. Recently, the INTERGROWTH-21st project established birth size charts based on low-risk newborns [14, 15]. These charts are considered to reflect growth in the absence of significant comorbidities and are suitable tools of growth assessment for newborns, which are recommended for global application [16]. However, there is still some debate about whether it is appropriate to adopt one standard for newborns in different populations. Although the INTERGROWTH-21st project believes that the population difference is not significant when the nutritional and health needs of pregnant women are met [17], other studies have shown that these environments do not fully explain population differences in birth weight [18, 19].

In China, existing growth standards for the assessment of birth size were established in 1988 [20]. Due to social development and improvements in medical technology and healthcare services [21, 22], it is suggested that these standards should be updated. In addition, national or regional monitoring data have demonstrated that there are differences in birth weight [22, 23] or head circumference [24] between Chinese and other populations. Therefore, the National Health Commission of China organized a special national study to update the birth size charts by gestational age. This paper will describe the difference in newborn birth size at the population level between Chinese and INTERGROWTH-21st populations, compare the new Chinese birth weight, length and head circumference charts by gestational age with INTERGROWTH-21st charts, and discuss their application in Chinese newborns by analyzing those differences in the prevalence of small for gestational age (SGA) or large for gestational age (LGA) based on Chinese and INTERGROWTH-21st birth weight charts.

## Methods

### Study design

The cross-sectional survey was prospectively conducted in nine cities, which included Beijing, Harbin, Xi'an, Shanghai, Nanjing, Wuhan, Fuzhou, Guangzhou and Kunming, from June 2015 to November 2018. In addition, the other four cities (Tianjin, Shenyang, Changsha and Shenzhen) surrounding the nine cities were included after July 2017 to add the number of early preterm newborns. Some maternal and child health hospitals or general hospitals in these cities that met the following conditions were selected: (1) the number of annual deliveries was > 1000; (2) there were both obstetrics and neonatal pediatrics departments; and (3) medical equipment in neonatal pediatric departments was adequate. A total of 69 hospitals from the 13 cities were selected.

### Subjects and sampling method

Subjects were live newborns from 24^+0^ to 42^+6^ weeks’ gestational age and their exclusion criteria were: (1) twins or multiple births; (2) unclear gestational age; (3) in vitro fertilization; (4) congenital malformation, limb defects, fetal edema or chromosomal abnormalities; (5) both or one of the parents is of non-Chinese origin; (6) mother's height < 145 cm; (7) mother's age < 18 or > 40 years old; (8) mothers who smoked, consumed alcohol or abused substances at three months prepregnancy or during pregnancy; (9) mothers who continued to take corticosteroids or other immunosuppressants during pregnancy for more than one month; (10) full-term newborns (37^+0^ to 42^+6^ weeks’ gestational age) whose mothers had some risk factors for fetal growth restriction, including severe anemia (hemoglobin ≤ 60 g/L), gestational diabetes, preeclampsia, eclampsia, hyperthyroidism or hypothyroidism, cardiorenal insufficiency, chronic hypertension; (11) preterm newborns (24^+0^ to 36^+6^ weeks’ gestational age) whose mothers had some significant risk factors for fetal growth restriction, including severe anemia (hemoglobin ≤ 60 g/L), gestational diabetes that was not effectively controlled by diet or exercise intervention, severe preeclampsia, eclampsia, severe cardiorenal insufficiency (cardiac function grade III or above and renal insufficiency decompensated stage or above), hyperthyroidism or hypothyroidism that could not be effectively controlled by drug therapy. It should be noted that the exclusion criteria of preterm infants were not as strict as those of full-term infants because of the limitation of the number of preterm infants who met these strict inclusion criteria, especially for those early preterm infants, as well as the fulfilling requirements on the sample size for constructing the growth charts as much as possible.

The gestational age was calculated in exact weeks combined with the mother's last menstrual period (LMP) and pregnancy ultrasound assessment within the first trimester. The LMP assessment was used when the two methods estimate differed by < 1 week; otherwise, the early pregnancy ultrasound assessment was used. Those subjects were divided into 19 groups in complete weeks from 24 to 42 weeks of gestation.

According to the statistical accuracy requirements for establishing growth charts, the sample size of each gestational age group should be generally at least 200 [25]. In this survey, considering the sample size requirements for establishing growth charts and the actual number of early preterm births, we required that the sample size was approximately 100 for 37–41 weeks of gestation and approximately 50 for 29–36 weeks of gestation by sex and gestational age group in each city, while for newborns under 29 weeks or 42 weeks of gestation, we tried our best to increase their collection during the investigation to ensure the accuracy of the extreme percentile.

Full-term newborns aged 37–41 weeks of gestation were sampled by stratified cluster sampling according to sex and gestational age group in the selected hospitals of each city. Moreover, those full-term newborns were evenly distributed by season by random sampling from each season. Because the number of newborns born at 42 weeks of gestation and preterm newborns who met the inclusion criteria was limited, all newborns born at these gestational ages in selected hospitals of each city during our survey period were included when they met the inclusion criteria to meet their sample size requirements. A total of 24,375 newborns were investigated.

### Measurements

Birth weight was measured within 12 hours of birth using a neonatal electronic weighing scale to the nearest 10 g. Birth length was measured within 24 hours of birth using an Infantometer to the nearest 0.1 cm. Head circumference was measured within 24 hours of birth using a flexible, nonstretchable plastic tape to the nearest 0.1 cm. All indicators were measured twice by two trained investigators according to the same standardized method [26], and the average value of the two measurements was calculated. Additional information on maternal and neonatal basic characteristics was obtained by questionnaire or consulting obstetrical medical records.

### Quality control

Measuring equipment for length and head circumference at all sites was uniformly equipped, and the neonatal electronic weighing scales of all the sites were qualified by the unified standardized weights before investigation. Standardized weights (10 g, 50 g, 100 g and 500 g) and steel rulers (accurate to 0.1 cm) served to calibrate the measuring equipment every week. It was required that the error not exceed 10 g for the electronic scale and 0.5 cm for the infantometer or nonstretchable plastic tape. Equipment whose error exceeded the range was corrected or replaced in a timely manner. All investigators had participated in rigorous specialized training and passed an examination before the investigation. Intraobserver and interobserver measurement errors were no more than 10 g for weight and 0.5 cm for length or head circumference. The same protocols and quality control methods were adopted across sites.

### Statistical analysis

Baseline data were analyzed using descriptive statistics in SPSS 21.0. The Generalized Additive Model for Location, Scale and Shape (GAMLSS) [27–29] was employed to create smoothed percentile curves from 24 to 42 weeks of gestation, which can be performed within the GAMLSS 4.3-1 library running under R 3.1.2. These curves were generated using the GAMLSS model with Box‒Cox *t* (BCT) distribution with cubic spline smoothing for birth weight and Box‒Cox power exponential (BCPE) distribution with cubic spline smoothing for birth length and head circumference according to the minimum value of global deviance, Akaike information criterion and Bayesian information criterion of the GAMLSS model among Box‒Cox Cole-Green, BCPE and BCT. The *Z* scores of measurements were calculated using the INTERGROWTH-21st standards (INTERGROWTH-21st-Newborn-tool-win20170217) [14, 15], and the one-sample *t* test method of *Z* scores of measurements was used to compare the difference between Chinese newborns and the INTERGROWTH-21st standards. The 10th and 90th percentiles of birth weight were taken as the cutoff points for defining small for gestational age (SGA, < 10th), appropriate for gestational age (AGA, 10–90th), or large for gestational age (LGA, > 90th). The proportions of SGA and LGA and their 95% confidence intervals (CIs) were calculated using both Chinese charts and INTERGROWTH-21st charts, and the consistency in the classification of newborns using the two charts was analyzed.

## Results

### Basic characteristics

Table 1 shows the basic characteristics of all the newborns and their mothers.

**Table 1:** Basic characteristics of the study population

Variables	All the newborns	Gestational age group (wk)			
		24–28	29–32	33–36	37–42
Sex of newborns, *n* (%)					
Male	13,197 (54.1)	586 (60.0)	2255 (58.4)	4201 (56.6)	6155 (50.8)
Female	11,178 (45.9)	390 (40.0)	1608 (41.6)	3224 (43.4)	5956 (49.2)
Maternal education or the years of maternal education, *n* (%)					
College or above (≥ 16 y)	15,044 (61.8)	506 (51.8)	1879 (48.6)	4194 (56.5)	8465 (69.9)
Senior school or technical secondary school (12–15 y)	5616 (23.0)	252 (25.8)	1173 (30.4)	1875 (25.3)	2316 (19.1)
Junior school or below (< 12 y)	3703 (15.2)	209 (21.4)	810 (21.0)	1355 (18.2)	1329 (11.0)
Missing	12 (0.0)	9 (0.9)	1 (0.0)	1 (0.0)	1 (0.0)
Maternal age (y), mean (SD)	31.5 (5.1)	32.8 (5.9)	32.5 (5.8)	31.8 (5.3)	30.9 (4.6)
Maternal height (cm), mean (SD)	161.0 (4.9)	160.4 (4.7)	160.4 (4.6)	160.6 (4.8)	161.5 (5.0)
Maternal BMI (kg/m^2^), mean (SD)	21.0 (3.0)	21.6 (3.2)	21.6 (3.0)	21.2 (3.1)	20.6 (2.8)
Maternal weight gain in pregnancy (kg), mean (SD)	13.8 (5.0)	9.6 (4.4)	11.4 (4.8)	13.2 (4.8)	15.2 (4.8)
Method of delivery, *n* (%)					
Vaginal delivery	14,950 (61.4)	689 (70.6)	1858 (48.1)	3781 (50.9)	8622 (71.2)
Cesarean delivery	9414 (38.6)	282 (28.9)	2002 (51.8)	3643 (49.1)	3487 (28.8)
Missing	11 (0.0)	5 (0.5)	3 (0.1)	1 (0.0)	2 (0.0)
Parity, *n* (%)					
Nulliparous	16,047 (65.9)	578 (59.2)	2076 (53.8)	4654 (62.7)	8739 (72.2)
Multiparous	8319 (34.1)	396 (40.6)	1782 (46.1)	2771 (37.3)	3370 (27.8)
Missing	9 (0.0)	2 (0.2)	5 (0.1)	0 (0.0)	2 (0.0)

*BMI* body mass index, *SD* standard deviation

### The updated Chinese growth charts

**Fig. 1:**
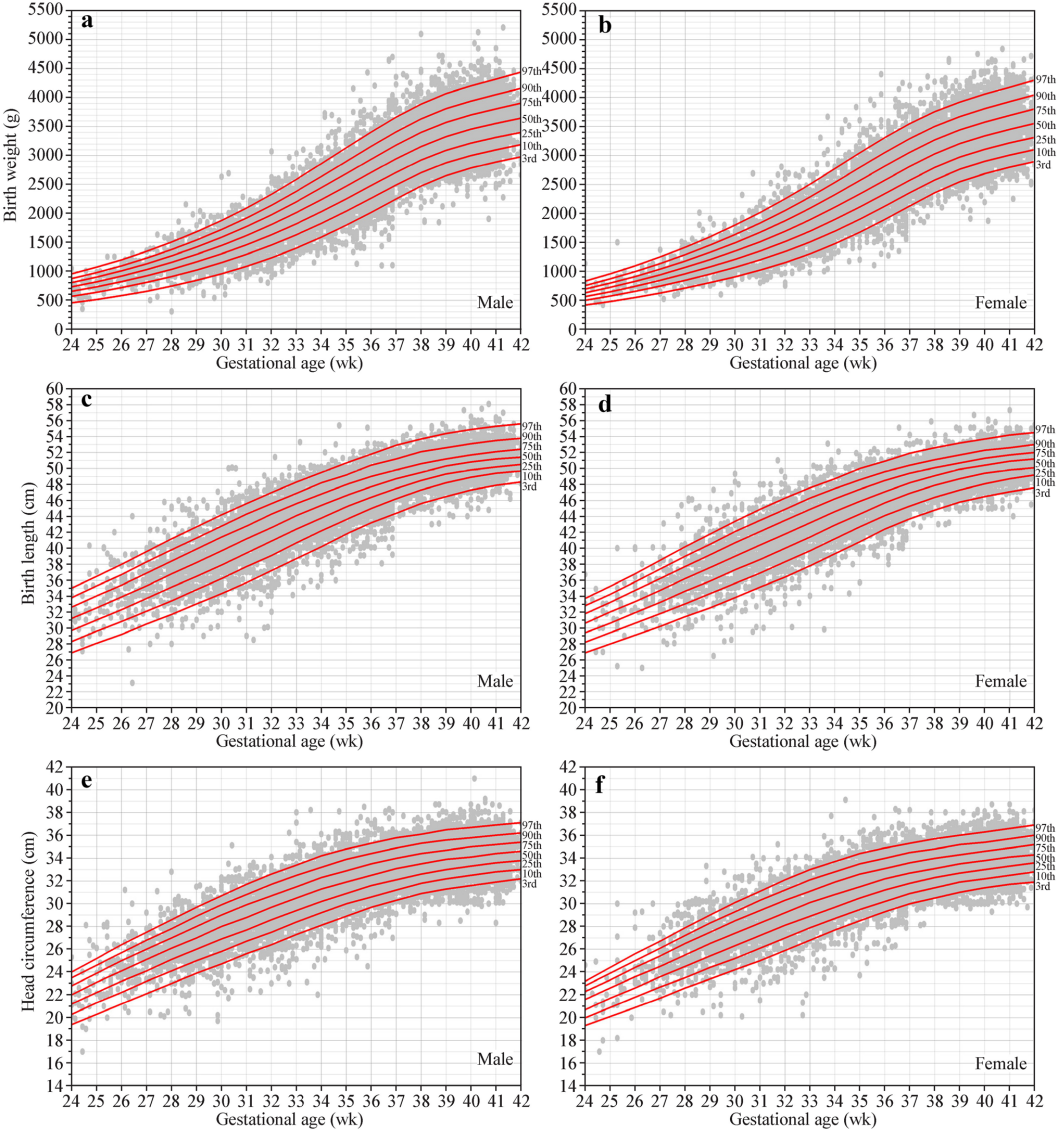
Fitted 3rd, 10th, 25th, 50th, 75th, 90th and 97th smoothed centile curves (red lines) for birth weight (**a**, **b**), birth length (**c**, **d**) and head circumference (**e**, **f**) according to gestational age (gray circles indicate the actual observations)

The smoothing fitted centile curves for birth weight, length and head circumference from 24 to 42 weeks of gestation for males and females and their fitness with the actual observation values are presented in Fig. 1.

### Comparison of the updated Chinese charts with the INTERGROWTH-21st charts [14, 15]

The *Z* scores of measurements in Chinese newborns in each gestational age group are shown in Table 2. In general, we found that the *Z* scores of birth weight and length of Chinese newborns were higher than 0, especially the *Z* scores of newborns at 37–40 weeks of gestation, which were up to 0.14–0.25 for birth weight and 0.54–0.74 for birth length. The *Z* scores of head circumference were not statistically significant in most gestational age groups.

**Table 2:** *Z* scores of birth weight, length and head circumference using the INTERGROWTH-21st standards

Gestational age (wk)	*n*	*Z* scores								
		Birth weight			Birth length			Head circumference		
		Mean	SD	*P*	Mean	SD	*P*	Mean	SD	*P*
24^+0^–24^+6^	41	0.05	1.33	0.801	− 0.29	1.08	0.096	− 0.48	1.55	0.056
25^+0^–25^+6^	57	0.55	0.84	< 0.001	0.02	1.18	0.920	0.15	1.46	0.449
26^+0^–26^+6^	119	0.39	0.70	< 0.001	− 0.25	1.18	0.024	0.17	1.17	0.118
27^+0^–27^+6^	242	0.30	0.88	< 0.001	− 0.19	1.13	0.008	0.10	1.19	0.207
28^+0^–28^+6^	517	0.32	0.93	< 0.001	− 0.09	1.11	0.060	0.28	1.19	< 0.001
29^+0^–29^+6^	632	0.15	0.92	< 0.001	− 0.19	1.11	< 0.001	0.04	1.17	0.391
30^+0^–30^+6^	853	0.13	0.91	< 0.001	− 0.07	1.06	0.062	0.17	1.16	< 0.001
31^+0^–31^+6^	1088	0.06	0.90	0.030	− 0.05	1.08	0.114	0.02	1.14	0.474
32^+0^–32^+6^	1290	0.06	0.91	0.027	− 0.01	1.02	0.828	− 0.03	1.18	0.325
33^+0^–33^+6^	1212	0.01	0.85	0.571	0.00	1.27	0.913	− 0.26	1.29	< 0.001
34^+0^–34^+6^	1658	− 0.05	0.85	0.015	0.10	1.21	0.001	− 0.11	1.18	< 0.001
35^+0^–35^+6^	1995	− 0.07	0.95	0.002	0.27	1.16	< 0.001	− 0.03	1.24	0.276
36^+0^–36^+6^	2560	− 0.04	0.95	0.034	0.41	1.13	< 0.001	0.00	1.19	0.863
37^+0^–37^+6^	1877	0.18	0.86	< 0.001	0.74	0.91	< 0.001	0.06	1.09	0.012
38^+0^–38^+6^	2444	0.25	0.88	< 0.001	0.67	0.85	< 0.001	0.11	1.11	< 0.001
39^+0^–39^+6^	2989	0.18	0.89	< 0.001	0.61	0.85	< 0.001	0.02	1.12	0.227
40^+0^–40^+6^	2757	0.14	0.90	< 0.001	0.54	0.87	< 0.001	− 0.12	1.17	< 0.001
41^+0^–41^+6^	1932	0.12	0.89	< 0.001	0.43	0.91	< 0.001	− 0.19	1.21	< 0.001
42^+0^–42^+6^	112	− 0.08	0.99	0.423	0.24	1.07	0.017	− 0.27	1.37	0.039
Total	24,375	0.10	0.91	< 0.001	0.35	1.06	< 0.001	− 0.02	1.18	0.006

**Fig. 2:**
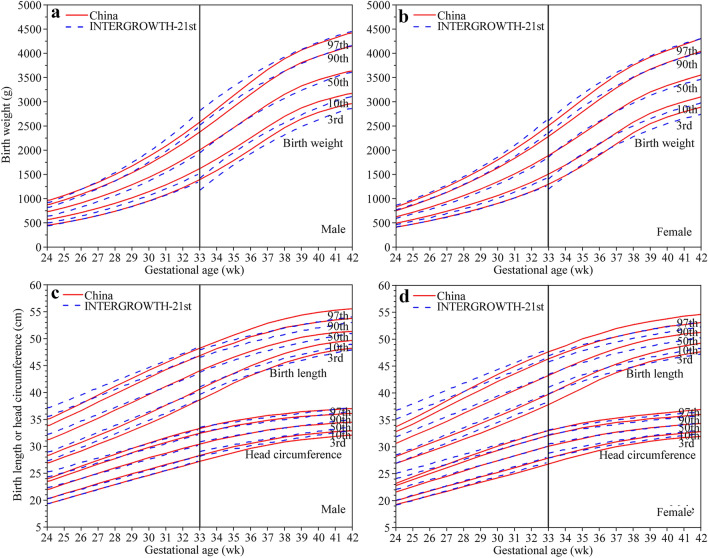
Comparison of centile curves for birth weight (**a**, **b**), length and head circumference (**c**, **d**) of Chinese newborns with those of the INTERGROWTH-21st standards

Figure 2 displays the differences in the Chinese birth size centile curves from the INTERGROWTH-21st charts. The 10th percentile of Chinese birth weight was 28–144 g higher than that of INTERGROWTH-21st, and a larger difference was observed at 38–41 weeks of gestation (79–144 g). The 50th percentile of Chinese birth weight was 29–92 g higher than that of the INTERGROWTH-21st except for 34–36 weeks of gestation, while the 90th percentile of Chinese birth weight was lower at 29–37 weeks of gestation (20–156 g). The 10th, 50th and 90th percentiles of Chinese birth length were 0.1–0.6 cm, 0.2–1.2 cm, and 0.1–2.3 cm shorter, respectively, than those of the INTERGROWTH-21st charts before 33 weeks of gestation and then gradually became higher; for example, the 10th, 50th and 90th percentiles at 38–41 weeks were 0.5–1.1 cm, 0.9–1.3 cm and 0.9–1.3 cm higher, respectively, than those of the INTERGROWTH-21st chart. The 10th percentile of head circumference was similar to that of the INTERGROWTH-21st charts before 33 weeks (the difference was 0.1–0.2 cm) and then 0.1–0.9 cm lower than that of the INTERGROWTH-21st charts. The difference in the 50th percentile of the head circumference was within 0.5 cm, and that of the 90th percentile after 28 weeks was similar (0.1–0.3 cm).

### Prevalence of SGA and LGA using both the Chinese and INTERGROWTH-21st birth weight charts and their consistency

**Fig. 3:**
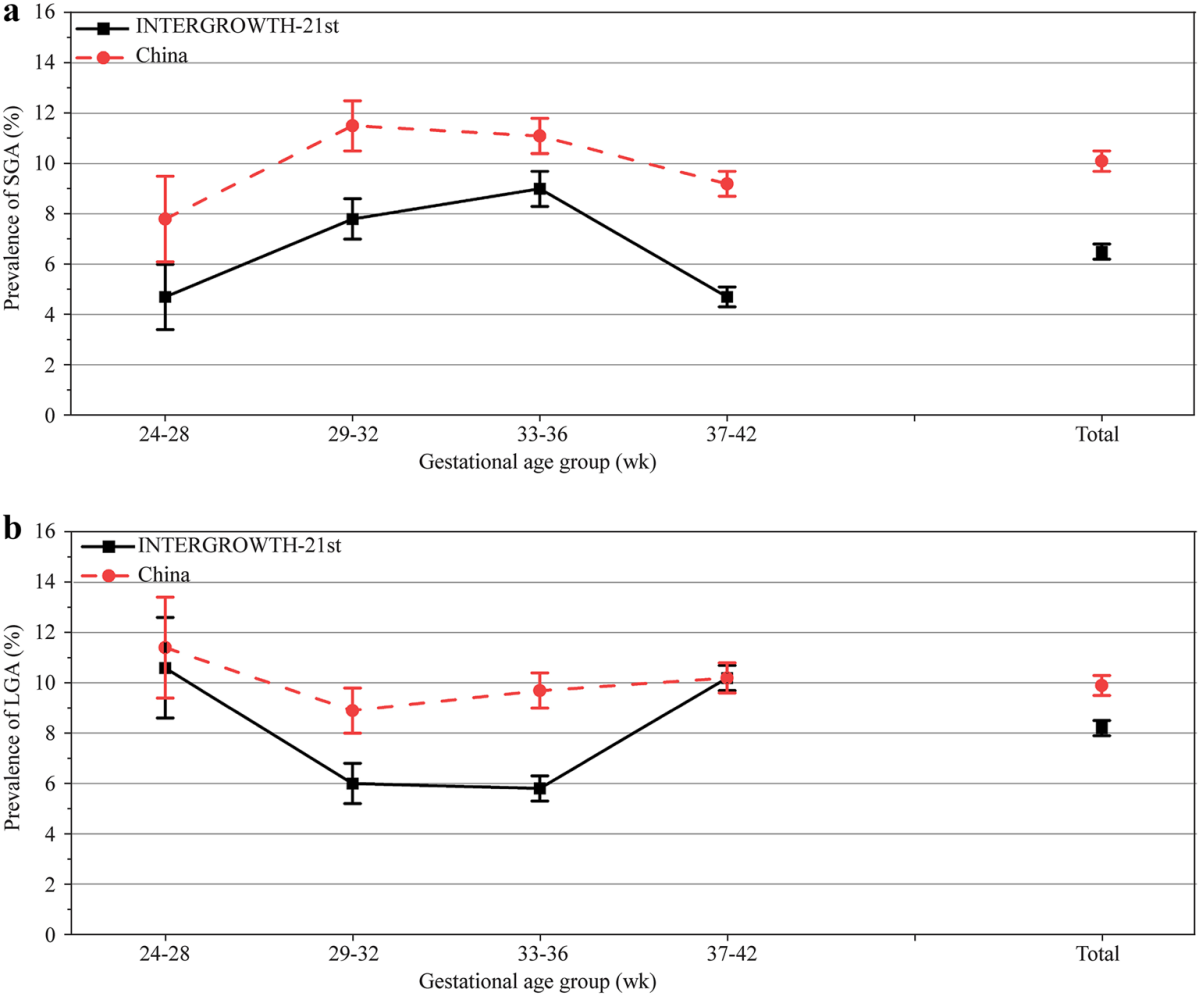
The prevalence of SGA and LGA and their 95% CI by Chinese charts and INTERGROWTH-21st charts. *SGA* small for gestational age, *LGA* large for gestational age, *CI* confidence interval

The prevalence of SGA was 10.1% (95% CI = 9.7%–10.5%) using the Chinese chart and 6.5% (95% CI = 6.2%–6.8%) using the INTERGROWTH-21st chart. The prevalence of LGA was 9.9% (95% CI = 9.5%–10.2%) using the Chinese charts and 8.2% (95% CI = 7.9%–8.6%) using the INTERGROWTH-21st charts. The prevalence of SGA and LGA using the two charts in different gestational age groups is shown in Fig. 3.

Table 3 illustrates that 93.8% of newborns had the same classification, and 1507 (6.2%) newborns were classified into different categories by the two charts. Almost all of the SGA newborns classified by the INTERGROWTH-21st were also classified as SGA by the Chinese chart (99.6%), whereas 42.2% of the SGA newborns classified by the Chinese chart were AGA classified by the INTERGROWTH-21st. Additionally, 98.2% of LGA newborns classified by the INTERGROWTH-21st were classified as LGA by the Chinese chart, whereas 18.8% of the LGA classified by the Chinese charts were not LGA classified by the INTERGROWTH-21st chart.

**Table 3:** Consistency of the classification of SGA, AGA and LGA using Chinese and the INTERGROWTH-21st charts

Gestational age group (wk)	China^a^	INTERGROWTH-21st^b^		
		SGA	AGA	LGA
Total	SGA	1027 (57.8)	749 (42.2)	0 (0.0)
	AGA	4 (0.0)	18,790 (99.7)	55 (0.3)
	LGA	0 (0.0)	703 (18.8)	3033 (81.2)
	Total	1031 (4.2)	20,242 (83.1)	3088 (12.7)
< 28	SGA	27 (47.4)	30 (52.6)	0 (0.0)
	AGA	0 (0.0)	712 (97.7)	17 (2.3)
	LGA	0 (0.0)	10 (5.3)	179 (94.7)
	Total	27 (2.8)	752 (77.1)	196 (20.1)
28 to < 33	SGA	208 (64.2)	116 (35.8)	0 (0.0)
	AGA	0 (0.0)	2907 (100.0)	0 (0.0)
	LGA	0 (0.0)	146 (23.3)	481 (76.7)
	Total	208 (5.4)	3169 (82.1)	481 (12.5)
33 to < 37	SGA	388 (71.7)	153 (28.3)	0 (0.0)
	AGA	4 (0.1)	5622 (99.9)	0 (0.0)
	LGA	0 (0.0)	465 (37.1)	790 (62.9)
	Total	392 (5.3)	6240 (84.1)	790 (10.6)
37 to 42	SGA	404 (47.3)	450 (52.7)	0 (0.0)
	AGA	0 (0.0)	9549 (99.6)	38 (0.4)
	LGA	0 (0.0)	82 (4.9)	1583 (95.1)
	Total	404 (3.3)	10,081 (83.3)	1621 (13.4)

Data are presented as *n* (%). *SGA* small for gestational age, *AGA* appropriate for gestational age, *LGA* large for gestational age. ^a^Classification of SGA, AGA and LGA using China charts; ^b^classification of SGA, AGA and LGA using the INTERGROWTH-21st charts

## Discussion

New birth size charts were established based on infants born after uncomplicated pregnancies from various geographical regions of China. These sites are all located in provincial capitals or municipal cities, whose altitude is in the range of 3–397 m above sea level except for Kunming (1891 m above sea level). The per capita GDP of these provinces where all the sites are located in 2018 is higher than the national average (￥84,350 vs. ￥64,644), and the perinatal mortality rate of these provinces was 2.38–5.74 per thousand births, and their low birth weight rate was 2.45%–5.51% [30]. The average birth weight in the nine main cities was 3380 g for males and 3260 g for females [31]. Furthermore, basic characteristics showed that 85% of newborns’ mothers attained a higher educational level, the means of maternal height and BMI were similar to Chinese urban women’s average level [32], and most of newborns were first birth and vaginal delivery. It is suggested that the birth size in this study can reflect the growth of Chinese newborns who received adequate antenatal care in good economic-social environments.

The INTERGROWTH-21st project has established birth size charts based on low-risk populations in eight countries, which are considered to represent ideal intrauterine growth [14, 15]. Subsequently, an increasing number of studies have focused on comparing the INTERGROWTH-21st standard with their local population data and its application in different populations [18, 19, 33]. In this study, we first analyzed the average birth size at the population level and found that the average birth weight of Chinese infants born after uncomplicated pregnancies was heavier than that of the INTERGROWTH-21st Newborn Cross-Sectional Study (NCSS) population. Although the differences in birth length under 33 weeks of gestation were not statistically significant, the length of Chinese newborns older than 33 weeks of gestation was higher than that of the INTERGROWTH-21st NCSS population. This shows that the birth size of infants born after uncomplicated pregnancies whose mothers are adequately cared for during pregnancy in economically developed areas in China and are adequately cared for during pregnancy exceeds the birth size of the INTERGROWTH-21st NCSS population, that is, the slightly heavier weight and longer length. Similar population differences were also found in some other studies [19, 33]. Additionally, it was noted that the sample size of the INTERGROWTH-21st charts at 24–32 weeks was small (*n* = 408), especially the sample size of each gestational age group under 28 weeks, which was even less than 10. Correspondingly, this study had a relatively large sample size (*n* = 4839) at 24–32 weeks. Due to the difference in the sample size, we still cannot confirm whether this difference in birth size at 24–32 weeks of gestation reflects the actual population difference. Additionally, the inclusion criteria of preterm infants and the model selection (especially the smoothing method) in our study were not the same as those in the INTERGROWTH-21st project, which may cause slight differences between them.

In clinical practice, the 10th and 90th percentiles of birth weight charts are generally used as the screening threshold for SGA or LGA. To further understand the significance of the difference in birth size between the Chinese population and the INTERGROWTH-21st NCSS population, we analyzed the centile curves of birth weight. The 10th percentile of the Chinese birth weight chart was higher than that of the INTERGROWTH-21st chart, while the 90th percentile at most gestational ages was lower than that of the INTERGROWTH-21st chart, especially at 29–37 weeks of gestational age. Using the two charts, we also found that the prevalence of SGA and LGA by the Chinese chart was higher than that of the INTERGROWTH-21st chart. In addition, almost all the SGA and LGA newborns classified by INTERGROWTH-21st were also SGA or LGA newborns classified by Chinese charts. Similar results were reported in another study from Guangdong Province of China, which showed that the rate of SGA and LGA by INTERGROWTH-21st was lower than that of the local birth weight curve (7.98% vs. 10.21% for SGA, 8.37% vs. 9.88% for LGA, respectively) [23]. It pointed out that the application of the INTERGROWTH-21st charts in Chinese newborns may lead to underestimating the rate of SGA or LGA.

Unquestionably, whether a growth chart is appropriate requires a comparison of the occurrence of short- or long-term adverse health outcomes of newborns who are screened by different charts. A study on the relationship between neonatal birth size and adverse perinatal outcomes found that the risk of adverse outcomes of SGA newborns classified by only the race-based birth weight customized standard but not the INTERGROWTH-21st standard was still significantly higher than that of non-SGA infants. This suggests that the INTERGROWTH-21st standard may not identify SGA newborns with a high risk of adverse outcomes, especially in a population with a larger maternal body size. It is thought that local population correction is necessary to avoid misclassification when applying the INTERGROWTH-21st standard [34]. Subsequently, a cohort study from 10 countries also found that the INTERGROWTH-21st standards failed to detect some stillbirth high-risk SGA babies compared with customized birth weight standards based on race and other factors, and they also believed that the various rates of SGA in different countries by the INTERGROWTH-21st standards were more related to the physiological variation among populations, and the global application of unified standards may not be appropriate [35]. In China, the applicability of the INTERGROWTH-21st chart still needs to be further verified in future studies by comparing the short-term or long-term health outcomes of SGA or LGA identified by the Chinese chart and the INTERGROWTH-21st chart.

There were some limitations: (1) due to the strict inclusion criteria and the limitation of the number of premature births and time of the special investigation, the sample size of early preterm newborns was small, which may have a certain impact on the extreme percentile; (2) the exclusion criteria of newborns in this study were determined based on the common causes of abnormal intrauterine growth as well as other similar international studies [14, 15]. These exclusion criteria may not include all the possible influencing factors on intrauterine growth, such as iatrogenic deliveries that have been mentioned recently [36]; (3) this study did not obtain data on postnatal health outcomes, so we cannot compare predictive performance on the health risks of high-risk newborns classified by the two charts and cannot supply some evidence on the clinical significance of the difference between the new Chinese charts and the INTERGROWTH-21st charts. These results in the study only described the difference in newborn birth size at the population level compared with the INTERGROWTH-21st standard. In the future, more research will be needed to evaluate the predictive performance of various neonatal charts on health outcomes to determine which charts are more suitable for clinical application in a specified population.

In conclusion, new birth size charts established based on infants born after uncomplicated pregnancies living in developed economic-social environments reflect the growth of Chinese infants born after pregnancies free from major complications. These growth charts were different from the INTERGROWTH-21st charts. Differences in the classification of newborns by the two charts should receive attention, and whether the application of INTERGROWTH-21st in Chinese newborns will lead to misclassification needs to be validated in future clinical practice.

## Data Availability

The datasets generated and analyzed during the current study are not publicly available due to limitations of ethical approval involving the subject data and anonymity, but are available from the corresponding author on reasonable request.
